# Disruption of Calcium Homeostasis in Human Spermatozoa: Implications on Mitochondrial Bioenergetics, ROS Production, Phosphatidylserine Externalization, and Motility

**DOI:** 10.3390/antiox15020213

**Published:** 2026-02-06

**Authors:** Anita Bravo, Ignacio Jofré-Fernández, Rodrigo Boguen, Raúl Sánchez, Fabiola Zambrano, Pamela Uribe

**Affiliations:** 1 Center of Translational Medicine-Scientific and Technological Bioresource Nucleus (CEMT-BIOREN), Faculty of Medicine, Universidad de La Frontera, Temuco 4810296, Chile; anita.bravo@ufrontera.cl (A.B.); raul.sanchez@ufrontera.cl (R.S.); fabiola.zambrano@ufrontera.cl (F.Z.); 2 Scientific and Technological Bioresource Nucleus, BIOREN, Universidad de La Frontera, Temuco 4811230, Chile; ignacio.jofre@ufrontera.cl; 3 Laboratorio de Investigación en Salud de Precisión, Departamento de Procesos Diagnósticos y Evaluación, Facultad de Ciencias de la Salud, Universidad Católica de Temuco, Temuco 4780000, Chile; rboguen@uct.cl; 4 Department of Preclinical Science, Faculty of Medicine, Universidad de La Frontera, Temuco 4781176, Chile; 5 Department of Internal Medicine, Faculty of Medicine, Universidad de La Frontera, Temuco 4781176, Chile

**Keywords:** oxidative stress, Ca^2+^ overload, energy metabolism, cell death

## Abstract

The etiology of male infertility is linked to oxidative stress, which is an imbalance caused by an excess of reactive oxygen species (ROS) that can negatively impact sperm function. It is known that a strong stimulus to induce excessive ROS production by spermatozoa is an intracellular calcium (Ca^2+^) overload; however, the link between Ca^2+^ dysregulation, ROS production, and impaired sperm function is still an area requiring further research. This investigation aimed to characterize the intracellular Ca^2+^ overload detrimental effects on human sperm quality. The intracellular Ca^2+^ overload was achieved by dose-dependent incubation with ionomycin, followed by analysis of key functional sperm parameters. Ca^2+^ overload caused an increase in cytosolic and mitochondrial ROS production, dissipation of mitochondrial membrane potential (ΔΨm), reduction in ATP content, cAMP levels, and motility. Furthermore, Ca^2+^ overload promoted phosphatidylserine externalization and a decrease in sperm viability. This study provides novel insights into the interplay between ROS and Ca^2+^ signaling, highlighting that disruption of homeostasis induces OS, leading to impairment of sperm quality. These findings not only contribute to the understanding of the mechanisms underlying male infertility but also provide an in vitro model for future research aimed at optimizing human sperm quality in patients with seminal OS.

## 1. Introduction

Infertility is a pathology of the male or female reproductive system [1,2] that affects millions of people worldwide, with devastating consequences that have a direct impact on the psychological well-being of couples struggling with infertility [3,4]. Currently, infertility is a highly prevalent condition affecting approximately 15% of couples trying to conceive worldwide [5]. The percentage of infertility attributable to the male partner ranges from 20 to 70% worldwide, with at least 30 million men worldwide being infertile [6].

At physiological levels, reactive oxygen species (ROS) act as signaling molecules that regulate processes such as motility, hyperactivation, capacitation, the acrosomal reaction, and fertilization capacity [7]. Among the factors that promote the impairment of sperm function and the subsequent development of male infertility, highlight oxidative stress (OS), which is characterized by excessive levels of ROS, that outweigh the antioxidant capacity [8]. Seminal ROS are produced primarily by leukocytes, but also by spermatozoa themselves, as a byproduct of oxidative metabolism, and through the action of specific enzymes called oxidases [9]. Several factors promote OS in the male gamete, including environmental and lifestyle conditions, which converge to ultimately induce an increase in ROS production by sperm cells and the development of OS [10]. OS is a key factor in the etiology of male infertility because it increases ROS levels, which damage sperm function [10]. The high content of polyunsaturated fatty acids in the sperm membrane makes them particularly vulnerable to ROS-mediated lipid peroxidation, which compromises membrane fluidity, [11] and impairs the ability to fuse with the oocyte [12]. Finally, this OS-mediated effect stimulates the cell death process [13].

Excessive ROS levels also cause damage to the nuclear and mitochondrial DNA [10] by oxidizing vulnerable bases, particularly guanines, which promotes the induction of DNA strand breaks [13]. Disruption of DNA integrity in mammalian spermatozoa is clinically relevant because it plays an important role in determining the subsequent developmental trajectory of the embryo [14]. These alterations highlight the link between OS and poor semen quality, emphasizing the importance of understanding the mechanisms by which OS causes impaired sperm function.

At the cellular level, calcium (Ca^2+^) is an intracellular second messenger that regulates key physiological functions in cells [15]. However, disruption of intracellular Ca^2+^ homeostasis affects cell survival and contributes to the pathogenesis of several diseases [16,17]. Intracellular Ca^2+^ signaling pathways are tightly interconnected with ROS dynamics, forming a bidirectional regulatory loop [17]. Mitochondrial Ca^2+^ overload can directly enhance ROS generation through multiple mechanisms, including increased metabolic activity, cytochrome c dissociation, cardiolipin peroxidation, and mitochondrial permeability transition pore (mPTP) opening. In turn, elevated ROS levels can further disrupt Ca^2+^ homeostasis by modulating Ca^2+^ channels and transporters, leading to sustained Ca^2+^ influx and mitochondrial dysfunction [18]. This self-amplifying feedback loop, if unregulated, exacerbates oxidative stress, compromises mitochondrial integrity, and activates cell-death signaling pathways, ultimately driving irreversible cellular damage [19,20].

In spermatozoa, intracellular Ca^2+^ overload is a potent stimulus for excessive ROS production [21]. Ca^2+^ plays a pivotal role in sperm function, contributing to several physiological processes that allow proper fertilization, including chemotaxis, sperm motility, capacitation, hyperactivation, the acrosome reaction, and the ability to bind to the zona pellucida [22,23]. However, dysregulation of intracellular Ca^2+^ signaling can lead to an impairment of sperm function [24]. Intracellular Ca^2+^ overload induced by the Ca^2+^ ionophore A23187 causes a reduction in motility and viability of sperm, as well as an increase in DNA fragmentation [25]. Similarly, a sudden increase in intracellular Ca^2+^ concentration ([Ca^2+^]_i_) mediated by endocrine disruptor chemicals desensitizes sperm to physiological ligands [26]. According to this, a previous study by our research group demonstrated that an increase in [Ca^2+^]_i_ induced in vitro by the Ca^2+^ ionophore ionomycin triggers the mitochondrial permeability transition process, which is associated with an increase in ROS levels, a decrease in mitochondrial membrane potential (ΔΨm), and DNA damage in human spermatozoa [21]. Thus, although there are reports on the effects of Ca^2+^ overload on sperm function, the link between Ca^2+^ dysregulation, ROS production, and impaired sperm function is still not fully understood. With this background, the investigation aimed to characterize intracellular Ca^2+^ overload detrimental effects on the quality of human spermatozoa.

## 2. Materials and Methods

### 2.1. Semen Collection and Analysis

Fifteen normozoospermic healthy donors, aged between 19 and 30 years, with no associated chronic diseases, provided semen samples obtained by masturbation. Prior to participating in the study, the donors agreed to participate in the research and signed an informed consent form, approved by the Scientific Ethics Committee at the Universidad de La Frontera. Semen sample collection and analysis were performed according to World Health Organization guidelines [27]. The swim-up technique was used to select the motile sperm population, which were then re-suspended in a human tubal fluid medium (HTF; [28]).

### 2.2. Induction of Ca^2+^ Overload in Human Spermatozoa

To assess Ca^2+^ overload, we used a Ca^2+^ ionophore ionomycin (catalog number I24222; Molecular Probes, Life Technologies, Carlsbad, CA, USA). Ionomycin facilitates the toxicity study associated with cytosolic Ca^2+^ overload in the context of an in vitro model [25]. Specifically, the Ca^2+^ ionophore ionomycin facilitates Ca^2+^ influx from the extracellular medium and release from intracellular stores, thereby inducing a sustained intracellular Ca^2+^ increase [29,30].

### 2.3. Analysis of Intracellular Ca^2+^ Overload Induced by Ionomycin in Human Sperm Cells

To determine optimal ionomycin concentrations and incubation times, spermatozoa were pre-loaded with 2.5 μmol/L of FLUO4-AM (Molecular Probes, Life Technologies, Carlsbad, CA, USA; catalog number F14201) for 45 min at 37 °C. FLUO4-AM was previously diluted to 1 mmol/L with DMSO containing Pluronic acid F-127 at 500 μmol/L (catalog number P2443; Sigma-Aldrich Inc., St. Louis, MO, USA). In wells of a microplate, 200 μL of HTF medium supplemented with the following ionomycin concentrations: 0.01, 0.1, 1, or 10 μmol/L were added. A control without ionomycin was also included. Then, 50 μL of cell suspension (5 × 10^6^ spermatozoa/mL) was added to each well. Experiments were performed in triplicate. The FLUO4-AM fluorescence signal was detected using a Synergy HTX multi-mode plate reader (Biotek, Winooski, VT, USA) over time in intervals of 15 s for 1 h at 37 °C. Fluorescent images were acquired in epifluorescent microscopy (Axio Scope A1-Zeiss) and their signal distribution was evaluated in ImageJ (Version 1.54m, with MBF plugins). Based on the results, 0.1, 1, and 10 μmol/L of ionomycin concentration and a 1 h incubation period at 37 °C were selected as treatments for subsequent experiments.

### 2.4. Analysis of the Effect of Intracellular Ca^2+^ Overload on ROS Production in Human Spermatozoa

Intracellular ROS production was assessed using dihydroethidium dye (DHE; catalog number D23107; Molecular Probes, Life Technologies, Carlsbad, CA, USA). DHE is able to enter cells and react with superoxide anion (O_2_^−^) to form a fluorescent product that binds strongly to DNA, called 2-hydroxyethidium. Cell viability was simultaneously monitored using SYTOX™ Green (Molecular Probes, Life Technologies, Carlsbad, CA, USA; catalog number S7020), was included. SYTOX™ Green is a stain that binds with high affinity to nucleic acids and selectively penetrates cells with compromised plasma membranes, while remaining excluded from intact, viable cells. Briefly, 2 × 10^6^ spermatozoa/mL previously incubated with ionomycin and untreated control were washed once by centrifugation at 500× *g* for 5 min. Sperm were then re-suspended in 1 mL of HTF and DHE (2 mmol/L), and SYTOX^®^ Green (50 μmol/L) was added and incubated for 15 min at 37 °C. Finally, cells were washed once and re-suspended in 500 μL of HTF. Flow cytometry was used to analyze ROS production and expressed as the mean fluorescence intensity (MFI) of DHE (for more details, see flow cytometry analysis below).

### 2.5. Analysis of the Effect of Intracellular Ca^2+^ Overload on Mitochondrial O_2_^−^ Production in Human Spermatozoa

MitoSOX red (catalog number M36008; Molecular Probes, Life Technologies, Carlsbad, CA, USA), a stain that enters exclusively mitochondria and reveals a red fluorescence after oxidation by O_2_^−,^ was used to assess mitochondrial O_2_^−^ (mROS) production. The MitoSOX red was used alongside the cell viability probe SYTOX™ Green (Molecular Probes, Life Technologies, Carlsbad, CA, USA; catalog number S7020). Briefly, 2 × 10^6^ spermatozoa/mL previously exposed to ionomycin and an untreated control were washed once, re-suspended in 1 mL of HTF, and incubated with MitoSOX (5 mmol/L), and SYTOX™ Green (50 μmol/L) for 20 min at 37 °C. Finally, the spermatozoa were washed twice and re-suspended in 300 μL of HTF. Flow cytometry was used to analyze mROS production and expressed as the MFI of MitoSOX red (for more details, see flow cytometry analysis below).

### 2.6. Analysis of the Effect of Intracellular Ca^2+^ Overload on ΔΨm in Human Spermatozoa

The ΔΨm evaluation was performed with tetramethylrhodamine methyl ester perchlorate (TMRM; catalog number T668; Sigma-Aldrich Inc., St. Louis, MO, USA). TMRM is a cell-permeable probe that acts as a red/orange fluorescent potentiometric reporter that accumulates within active mitochondria [31] in direct proportion to the state of ΔΨm [32]. Briefly, 1 × 10^6^ spermatozoa/mL previously exposed to ionomycin and an untreated control were washed once, re-suspended with 1 mL of HTF, and incubated with TMRM (250 μmol/L) and SYTOX^TM^ Green (50 μmol/L) for 25 min at 37 °C. Finally, sperm cells were washed twice and re-suspended in 300 μL of HTF. Flow cytometry was used to analyze ΔΨm and expressed as the MFI of TMRM (for more details, see flow cytometry analysis below).

### 2.7. Analysis of the Effect of Intracellular Ca^2+^ Overload on ATP Levels in Human Spermatozoa

ATP Determination kit (Molecular Probes, Life Technologies, Carlsbad, CA, EUA; catalog number A22066), was used to assess the ATP levels according to the manufacturer’s instructions. Briefly, 5 × 10^6^ spermatozoa/mL previously exposed to ionomycin and an untreated control, were washed once and re-suspended in 1 mL of HTF. Then, 10 μL of each sperm suspension was added to a white-walled 96-well luminometer plate, containing 100 μL of the standard reaction solution, and incubated for 60 s at 25 °C. Finally, a luminometer (Luminoskan, Thermo Scientific, Atlanta, GA, USA) was used to measure the relative luminescence units (RLU) at 100 ms. The background luminescence obtained in the control corresponded to each determination. The ATP concentration was obtained by extrapolation in a calibration curve (0–100 μmol). Results of ATP content were expressed as μmol per × 10^6^ cells.

### 2.8. Analysis of the Effect of Intracellular Ca^2+^ Overload on cAMP Content in Human Spermatozoa

Evaluation of cAMP content was performed using the kit cAMP-Screen Direct (Molecular Probes, Life Technologies, Carlsbad, CA, EUA; Catalog number 4412187). One mL of sperm suspension at 5 × 10^6^ spermatozoa/mL was exposed to ionomycin treatments, and a control well was also included in the incubation time. Then, the samples were centrifuged at 900× *g*, 5 min. The resulting pellet was processed according to the manufacturer’s protocol. A luminometer (Luminoskan, Thermo Scientific, Atlanta, GA, USA) was used to measure the RLU, and the corresponding cAMP values were extrapolated from a standard curve generated with cAMP standards between 0 and 6 pmol/L. The cAMP content was calculated as pmol per 10^6^ cells (pmol × 10^6^ cells).

### 2.9. Analysis of the Effect of Intracellular Ca^2+^ Overload on Sperm Motility in Human Spermatozoa

A computer-aided sperm analysis (CASA) system with the Integrated Sperm Analysis System software version 1 (ISAS; Proiser, Valencia, Spain) was used to evaluate sperm motility. At least 200 sperm were analyzed using negative contrast for each test. The following parameters were set to classify a sperm as motile: 25 frames/s; 15–50 mm^2^ for sperm head area; and curvilinear velocity (VCL) 10 mm/s to classify a spermatozoon as motile [33]. Briefly, 5 × 10^6^ spermatozoa/mL were exposed to ionomycin treatment. An untreated control was also included. Then, using a microscope with a stage tempered at 37 °C, a minimum of 100 spermatozoa from at least four different fields were analyzed. The progressive motility percentage, non-progressive motility, and static spermatozoa were determined for each experiment.

### 2.10. Analysis of the Effect of Intracellular Ca^2+^ Overload on Phosphatidylserine (PS) Externalization in Human Spermatozoa

Annexin V-Alexa Fluor™ 488-conjugated (catalog number A13201; Molecular Probes, Life Technologies, Carlsbad, CA, EUA) was used to evaluate PS externalization in combination with propidium iodide (PI; catalog number P3566; Molecular Probes, Life Technologies, Carlsbad, CA, EUA) to exclude dead cells. For this, 1 × 10^6^ spermatozoa/mL were exposed to the previously established experimental conditions. An untreated control was also included. After incubation, spermatozoa were washed once and re-suspended in 100 μL of 1× annexin binding buffer. Then, 2 μL of Annexin V-Alexa Fluor^®^ 488 and PI (1 μmol/L) were added to the spermatozoa, and incubated for 15 min at room temperature. Finally, 400 μL of 1× Annexin binding buffer was added. Flow cytometry was then used to analyze the PS externalization, and the results were expressed as the percentage of spermatozoa positive for annexin V and negative for PI (Annexin V^+^/PI^−^).

### 2.11. Analysis by Flow Cytometry

The BD FACSCanto II flow cytometer (Becton Dickinson) controlled by BD FACSDiva™ v. 6.1.3 software (Becton Dickinson) was used to perform fluorescence analysis. Cells were analyzed at an aspiration rate of 60 µL/min, recording a total of 10,000 events. Fluorophores were excited with a 488 nm argon laser, and their detection was performed in the following channels: 585/42 nm channel (PE): PI, DHE, TMRM, and MitoSOX 530/30 nm channel (FITC): SYTOX™Green and Alexa Fluor^®^ 488. All on logarithmic scales.

### 2.12. Statistical Analysis

GraphPad Prism software package version 5 (GraphPad, La Jolla, CA, USA) was used to perform statistical analysis. All data were tested for normality using the D’Agostino’s test, and numerical results that failed the normality test were transformed to a logarithmic scale. A two-way ANOVA followed by a Bonferroni post-test was used to evaluate the effect of time and ionomycin concentration on [Ca^2+^]_i_, comparing the treatment with different concentrations of ionomycin versus the untreated control at each incubation time. One-way analysis of variance (ANOVA) with Dunnett’s post-test was used for statistical analysis of cytosolic ROS and mROS production, ΔΨm, ATP levels, cAMP content, PS externalization, viability, and Ca^2+^ overload during 1 h. To evaluate the total sperm motility, statistical analysis, a one-way ANOVA for nonparametric data (Kruskal–Wallis test) with Dunn’s post-test, was used. The analysis between the correlation of cytosolic ROS, mROS production, and intracellular Ca^2+^ overload was performed with parametric tests (Pearson product–moment correlation and Student–Newman–Keuls one-way analysis of variance). The results were expressed as mean ± SD, with a *p*-value less than 0.05 considered statistically significant. The experiments of ionomycin-induced Ca^2+^ overload were carried out in triplicate on different days using different semen samples. All experiments of flow cytometry were carried out in duplicate, on different days and using different semen samples.

## 3. Results

### 3.1. Intracellular Ca^2+^ Overload Induced by Ionomycin in Human Sperm Cells

**Figure 1:**
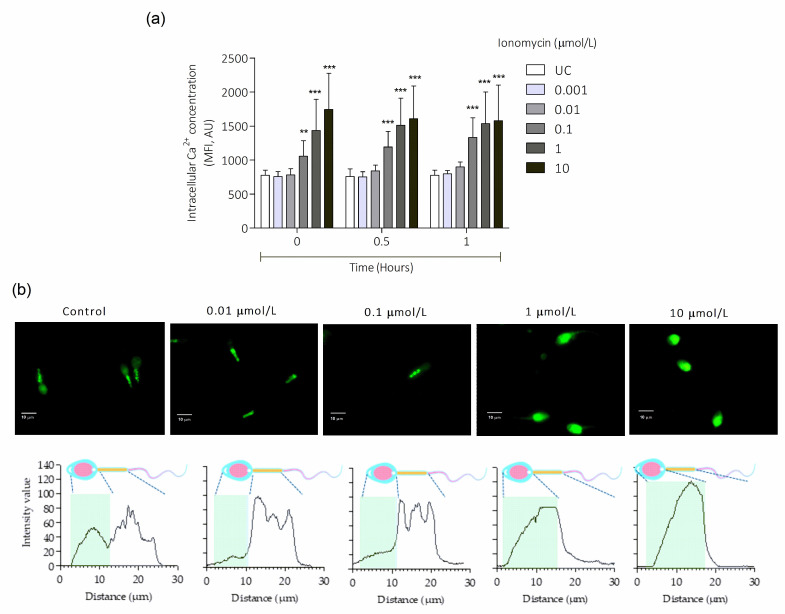
Intracellular Ca^2+^ overload induced by ionomycin in human spermatozoa. (**a**) Analysis of intracellular Ca^2+^ overload using different concentrations and exposure times of ionomycin. Spermatozoa were incubated with 0.001, 0.01, 0.1, 1, and 10 µmol/L. (**b**) Representative images of FLUO-4AM distribution in sperm cells (top row), and histograms depicting the distribution of fluorescence from head to tail (bottom rows); peaks correspond to the higher Ca^2+^ localization. Statistically significant differences compared to the UC group are indicated as ** *p* < 0.01, *** *p* < 0.001, N = 5. UC, untreated control; AU, arbitrary units; MFI, mean fluorescence intensity.

Analysis of intracellular Ca^2+^ overload in ionomycin-treated spermatozoa was performed at intervals of 15 s for 1 h in a multi-mode plate reader; however, in order to simplify the presentation of results, three incubation times (0, 0.5, and 1 h) were selected to perform the statistical analysis. The results showed a dependent response regarding concentration (*p* < 0.0001) and time (*p* < 0.0001). When spermatozoa were incubated for 1 h with lower ionomycin concentrations (0.001 and 0.01 µmol/L), no increase in Ca^2+^ overload was observed. However, a significant increase in FLUO-4AM intensity was observed at 0.1, 1, and 10 µmol/L compared to the untreated control at each incubation time (Figure 1a), and the maximal increase was observed at 10 µmol/L. The increase in Ca^2+^ overload with the higher ionomycin concentrations (0.1, 1, and 10 µmol/L) started at the beginning of incubation and continued with a steady increase up to 1 h of incubation. Thus, 0.1, 1, and 10 μmol/L of ionomycin concentrations and an incubation time of 1 h at 37 °C were selected for further analysis. Representative images of Ca^2+^ localization in sperm cells showed an intracellular Ca^2+^ signal from the middle piece to the head (Figure 1b). In the untreated control, the head and midpiece showed high fluorescence intensity of the probe, both separated by the neck. In the presence of 0.01 µmol/L, a decrease in signal from the head was observed, but an increase in midpiece was found. With 0.1 µmol/L, an increase in the signal near the neck but low to the head, with 1 µmol/L, a high intensity of the head and a decrease in the midpiece was observed. Finally, a complete increase in fluorescence intensity of the head and a total decrease in the midpiece were observed in the presence of 10 µmol/L. 

### 3.2. Intracellular Ca^2+^ Overload Effect on Cytosolic and Mitochondrial ROS Production in Human Spermatozoa

**Figure 2:**
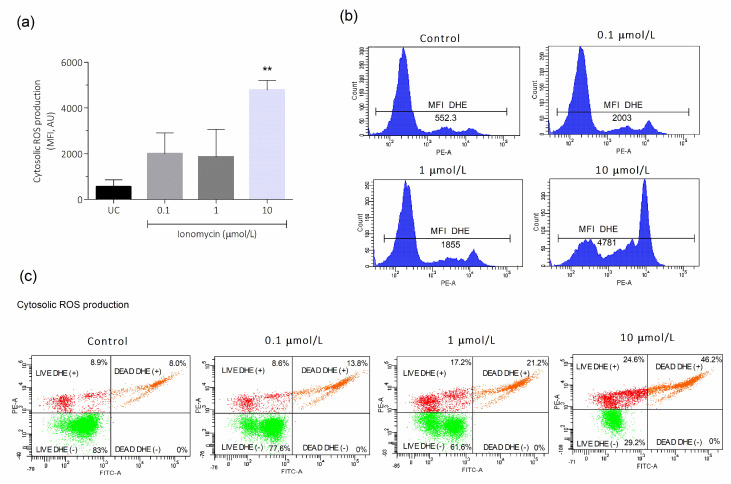
Effect of intracellular Ca^2+^ overload on cytosolic ROS production in human spermatozoa. The sperm cells were incubated for 1 h at 37 °C with 0.1, 1, and 10 μmol/L of ionomycin. After this, (**a**) cytosolic ROS production was evaluated by DHE/SYTOX™ Green by flow cytometry in the total sperm population (including live and dead cells). Three experiments were performed, and the results are presented as the mean ± SD. (**b**) Representative histograms of the MFI of DHE in the total sperm population from the flow cytometric analysis of one experiment. (**c**) Representative dot plots images from flow cytometry analysis of a single sperm sample, showing live and dead spermatozoa percentage with high and low ROS production (DHE+ and DHE−, respectively). ** *p* < 0.01. UC, untreated control; AU, arbitrary units; MFI, mean fluorescence intensity.

The effect of intracellular Ca^2+^ overload on cytosolic and mitochondrial ROS production was then evaluated. In the total sperm population (including live and dead cells), a statistically significant increase in cytosolic ROS production was observed after incubation with 10 μmol/L ionomycin compared to the untreated control (4781 ± 422.6 and 552.3 ± 305.3, respectively; *p* < 0.01, Figure 2a). Cytosolic ROS production histogram images analyzed by flow cytometry on the sperm population are depicted in Figure 2b, showing an increase in mean fluorescence intensity of DHE as the concentration of ionomycin increases. Also, dot plot images of the analysis, showing the percentage of live and dead spermatozoa with high and low ROS production (DHE+ and DHE-, respectively), are shown in Figure 2c. It is observed that ROS levels increased both in live and dead sperm as ionomycin concentration increases (Figure 2c), highlighting that the analysis of ROS levels should be performed on the sperm population, including live and dead cells, in order not to underestimate the total ROS production.

**Figure 3:**
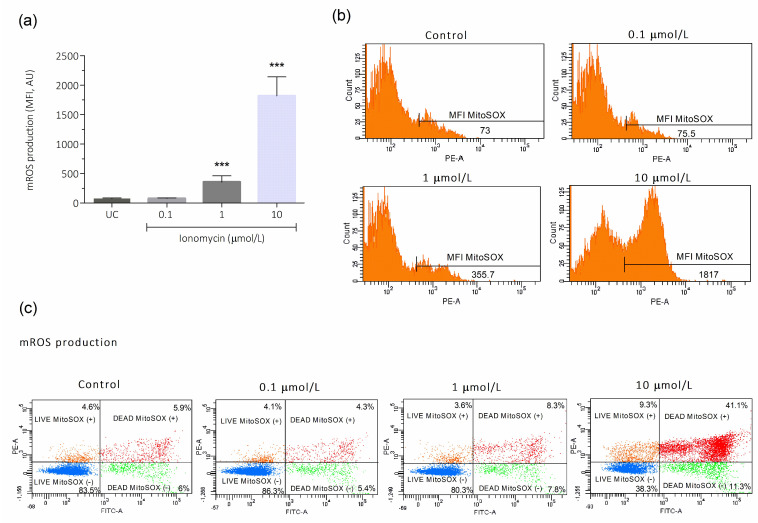
Effect of intracellular Ca^2+^ overload on mitochondrial ROS production in human spermatozoa. For all experiments, the sperm cells were incubated for 1 h at 37 °C with 0.1, 1, and 10 μmol/L of ionomycin. After this, (**a**) mitochondrial O_2_^−^ (mROS) production was evaluated by MitoSOX red/ SYTOX™ Green by flow cytometry in a total sperm population. Four different experiments were performed, and the results are presented as the mean ± SD. (**b**) Representative histograms of the MFI of MitoSOX in the total sperm population from the flow cytometric analysis of one experiment. (**c**) Representative dot plots images from flow cytometry analysis of a single sperm sample. *** *p* < 0.001. UC, untreated control; AU, arbitrary units; MFI, mean fluorescence intensity.

The mROS production results on the total sperm population showed an increase in O_2_^−^ production after incubation with 1 and 10 μmol/L of ionomycin (355.8 ± 106.5 and 1817 ± 322.4, respectively), compared to the untreated control (73.00 ± 14.49; *p* < 0.001; Figure 3a). Representative histogram images of mROS production on the total sperm population are depicted in Figure 3b, showing an increase in mean fluorescence intensity of MitoSOX with concentrations of 1 and 10 μmol/L of ionomycin. Also, the analysis of the dot plot images, showing the percentage of live and dead spermatozoa with high and low mROS production, is shown in Figure 3c. mROS increased both in live and dead sperm as ionomycin concentration increases (Figure 3c), which is more accentuated in dead sperm, suggesting that mitochondrial ROS production triggers sperm cell death.

### 3.3. Intracellular Ca^2+^ Overload Effect on ΔΨm and ATP in Human Spermatozoa

**Figure 4:**
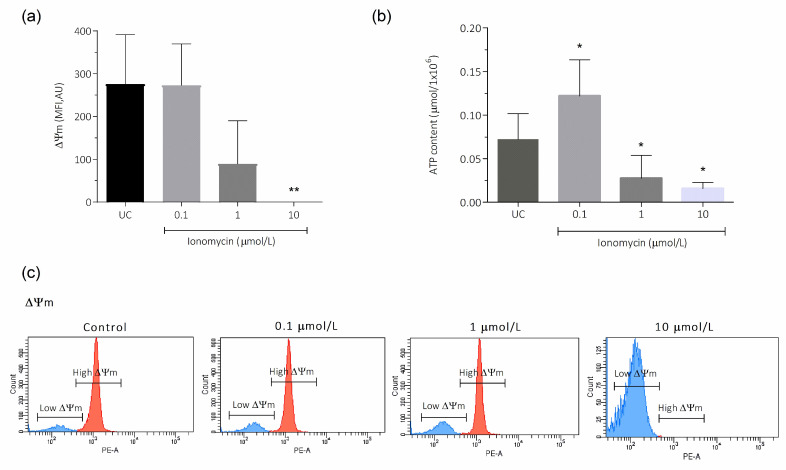
Intracellular Ca^2+^ overload effect on ΔΨm and ATP in human spermatozoa. For all experiments, the spermatozoa were incubated for 1 h at 37 °C with 0.1, 1, and 10 μmol/L ionomycin. (**a**) TMRM staining was used to evaluate ΔΨm in the total sperm population by flow cytometry. Five different experiments were performed, and the results are presented as the mean ± SD. (**b**) The ATP levels were evaluated in a total sperm population by a bioluminescence assay method. Five different experiments were performed, and the results are presented as the mean ± SD. (**c**) The images correspond to representative flow cytometry histograms of TMRM analysis on live sperm from one experiment. * *p* < 0.05; ** *p* < 0.01, N = 5. UC, untreated control; MFI, mean fluorescence intensity; AU, arbitrary units.

Mitochondria play a fundamental role in the generation of ATP through oxidative metabolism, regulating cellular metabolism. In this sense, we evaluated the ΔΨm and ATP content after induction of intracellular Ca^2+^ overload with ionomycin. The ΔΨm in live spermatozoa was significantly decreased after treatment with 10 μmol/L (0.00 ± 0.00), compared to the untreated control (276.3 ± 114.9; *p* < 0.01; Figure 4a). When ATP levels were evaluated, at 0.1 μmol/L ionomycin a significant increase in ATP content was observed (0.12 ± 0.04), compared to the untreated control (0.07 ± 0.02; *p* < 0.05; Figure 4b); however, this parameter was reduced with higher ionomycin concentrations, being statistically significant after treatment with 1 and 10 μmol/L (0.03 ± 0.03 and 0.02 ± 0.01, respectively), when compared to the untreated control (0.07 ± 0.02; *p* < 0.05; Figure 4b). Figure 4c shows representative flow cytometry histograms of ΔΨm analysis on live spermatozoa exposed to different concentrations of ionomycin, including the untreated control.

### 3.4. Effect of Intracellular Ca^2+^ Overload on cAMP Content in Human Spermatozoa

**Figure 5:**
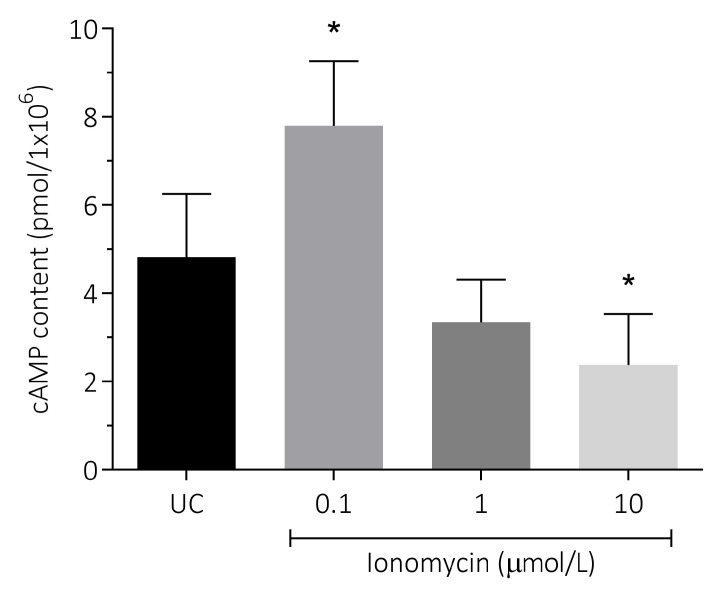
Effect of intracellular Ca^2+^ overload on cAMP content in human spermatozoa. Human spermatozoa were exposed for 1 h at 37 °C to 0.1, 1, and 10 µmol/L of ionomycin. Four different experiments were performed, and the results are presented as the mean ± SD. cAMP content was measured in a total sperm population by a chemiluminescent immunoassay. * *p* < 0.05, N = 4. UC, untreated control.

The effect of intracellular Ca^2+^ overload stimulated by ionomycin was evaluated on cAMP content as a mediator of important physiological sperm parameters. The results showed a significant increase in this parameter after incubation with 0.1 μmol/L of ionomycin (7.79 ± 1.47) compared to the untreated control (4.81 ± 1.43), while a decrease was observed at higher concentrations of ionomycin, which was statistically significant at 10 μmol/L (2.37 ± 1.16) compared to the untreated control (4.81 ± 1.43; *p* < 0.05; Figure 5).

### 3.5. Effect of Intracellular Ca^2+^ Overload on Sperm Motility in Human Spermatozoa

To evaluate the influence of intracellular Ca^2+^ overload on a critical parameter of sperm functionality, sperm motility was measured. The results showed a decrease in progressive motility after incubation with 10 μmol/L of ionomycin (0.0% ± 0.0) compared to the untreated control (30.6% ± 10.5; *p* < 0.05; Table 1). Non-progressive motility decreased after incubation with 1 and 10 μmol/L ionomycin (15.5% ± 15.8 and 0.0% ± 0.0, respectively) compared to the untreated control (47.2% ± 8.5; *p* < 0.05, *p* < 0.01; Table 1). This decrease pattern was observed in the rapid population, which reached 0%, and the immotile population, which increased to 100% at 10 μmol/L ionomycin (Table 1).

### 3.6. Intracellular Ca^2+^ Overload Effect on PS Externalization and Viability in Human Spermatozoa

**Figure 6:**
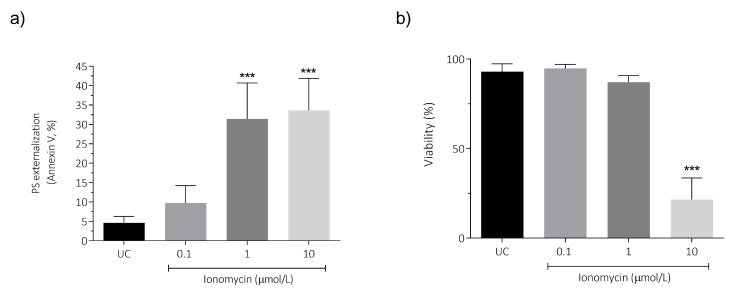
Effect of intracellular Ca^2+^ overload on (**a**) PS externalization and (**b**) sperm viability in human spermatozoa. For all experiments, the sperm cells were incubated for 1 h at 37 °C with 0.1, 1, and 10 µmol/L of ionomycin. Four different experiments were performed, and the results are presented as the mean ± SD. PS externalization in viable cells was evaluated using Annexin V/PI staining by flow cytometry. PI: propidium iodide. *** *p* < 0.001, N = 4. UC, untreated control.

Ca^2+^ overload induces various forms of cell death, including apoptotic cell death [34,35,36]. PS externalization was analyzed as an apoptotic marker along with viability. When human spermatozoa exposed to 1 and 10 μmol/L for 1 h were compared with the untreated control (4.61 ± 1.70; *p* < 0.001; Figure 6a), a significant increase in PS externalization (31.43% ± 9.28 and 33.63 ± 8.27, respectively) was observed. The PS externalization increase was associated with a decrease in sperm viability using 10 μmol/L of ionomycin (21.56% ± 12.00) compared to the untreated control (92.98% ± 4.34; *p* < 0.001; Figure 6b).

### 3.7. Correlation Between Intracellular Ca^2+^ Overload with Cytosolic ROS and mROS Production in Human Spermatozoa

**Figure 7:**
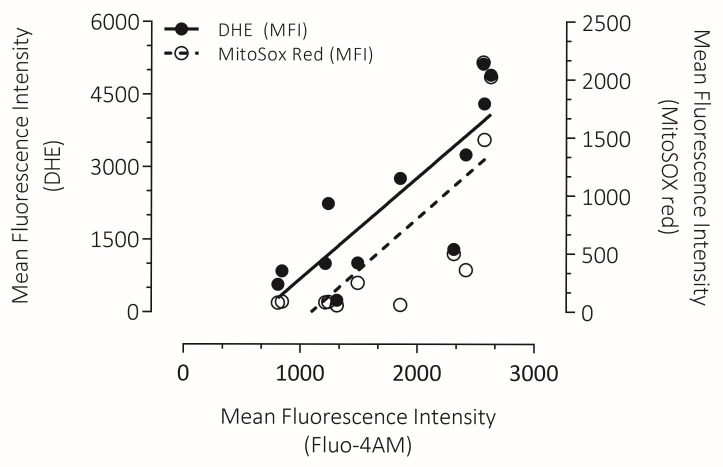
Correlation between cytosolic ROS production (DHE), mROS production (MitoSox Red), and the intracellular Ca^2+^ overload (Fluo-4 AM) induced by ionomycin in human spermatozoa. The correlation analysis of intracellular Ca^2+^ overload and cytosolic and mROS production was performed on the total sperm population. Both oxidative parameters showed a positive correlation with respect to Ca^2+^ overload (DHE; r= 0.8365, MitoSOX red; r = 0.7766) (*p* < 0.001).

Considering that it has been established that Ca^2+^ overload stimulates the generation of ROS [37], intracellular Ca^2+^ overload, and cytosolic and mROS production were evaluated on the total sperm population in order to analyze their correlation. We observed a positive correlation between intracellular Ca^2+^ overload and intracellular ROS production and mROS production. According to this correlation analysis, the increase in ionomycin concentration and subsequent Ca^2+^ overload leads to an increase in intracellular oxidation (r = 0.8365), and the O_2_^−^ production by mitochondria (r = 0.7766) (Figure 7).

## 4. Discussion

The Ca^2+^ relevance as a second messenger in signal transduction events is due to its spatiotemporal capacity to transmit information at the cellular level [38], a property that is strictly regulated through several cellular mechanisms. In sperm cells, Ca^2+^ is involved in the signaling to induce and modulate several important physiological processes, which are important for the proper fertilization of the oocyte [39]. Although there are some studies linking intracellular Ca^2+^ overload with impaired sperm function, the mechanisms underlying this impairment are not fully understood. In this sense, we evaluated the effect of intracellular Ca^2+^ overload induced by different concentrations of ionomycin on several parameters related to sperm function and metabolism in human spermatozoa.

First, we observed oscillations in Ca^2+^ localization in spermatozoa after exposure to different concentrations of ionomycin. Depending on the ionomycin concentration used, these oscillations were observed in the head and midpiece. This indicates the ability of human spermatozoa to regulate the efflux or influx of Ca^2+^ from intracellular stores in response to intracellular Ca^2+^ overload [40,41]. [Ca^2+^]_i_ signaling is a key regulatory mechanism in sperm function. There is considerable evidence that Ca^2+^ stored in intracellular organelles, such as the acrosome and mitochondria, is also functionally important in mammalian spermatozoa [42]. Indeed, the acrosome and mitochondria are pivotal places where sperm regulate [Ca^2+^]_i_ [43]. These findings confirm that acrosome- and mitochondrial-mediated Ca^2+^ signaling is essential for regulating Ca^2+^ concentration and plays a central role in controlling cellular behavior and function in mammalian sperm [44].

Our results showed that the increase in intracellular Ca^2+^ overload caused by exposure to low concentrations of ionomycin (0.1 µmol/L) was accompanied by an increase in ATP levels and cAMP content, without altering cytosolic and mROS production, ΔΨm, viability, and PS externalization. These results suggest that the Ca^2+^ increase induced by 0.1 µmol/L ionomycin has non-pathological effects on human spermatozoa, since the stimulation does not affect sperm quality. In addition, the lower concentration of ionomycin caused an increase in ATP and cAMP levels, which are related to processes important for sperm function, such as motility, capacitation, and hyperactivation [45]. These results suggest that the Ca^2+^ increase induced by 0.1 µmol/L ionomycin has non-pathological effects on human spermatozoa, preserving mitochondrial function and sperm quality and function. This can be explained by the fact that, under normal conditions, Ca^2+^ released into the cytosol can stimulate pathways that trigger a cellular response, including Ca^2+^ uptake by mitochondria, to accelerate ATP production [17]. Specifically, mitochondrial Ca^2+^ uptake modulates energy production by promoting the supply of NADH to the electron transport chain and by enhancing the activity of F1-Fo ATP synthase, thereby increasing NADH consumption [46,47]. Therefore, an increase in cellular bioenergetics mediated by Ca^2+^ is capable of increasing energy production from mitochondria to meet the energy demands of the cell [17], which may explain the results observed in this study. On the other hand, the Ca^2+^-mediated increase in ATP levels observed in this study can be translated into the maintenance of sperm motility [45,48]. In addition, the positive relationship between ATP production and sperm motility may be favored by the increase in cAMP, since this second messenger acts as a mediator with a positive influence on motility through the activation of the sperm Na^+^/H^+^ exchanger (sNHE) [49], which, together with the proton channel (HV) activation, could increase the pH_i_ and activate the CatSper channel [50,51], which is responsible for increasing the [Ca^2+^]_i_ and thus the production of ATP and the flagellar beating, promoting the hyperactivation of motility.

On the other hand, our results showed that the sustained increase in intracellular Ca^2+^ overload caused by the exposure of human spermatozoa to higher concentrations of ionomycin (1 and 10 µmol/L) caused deleterious effects on sperm cells, including an increase in cytosolic and mROS production, a decrease in ΔΨm, ATP, cAMP levels and motility while triggering PS externalization and decreasing sperm viability. These results suggest that the Ca^2+^ increase induced by 1 and 10 µmol/L ionomycin has a pathological effect on human spermatozoa, causing a detrimental effect on mitochondrial function and sperm quality. Regarding cytosolic ROS and mROS production, it has been observed that there is a positive correlation between the increase in intracellular Ca^2+^ overload caused by ionomycin exposure and the increase in cytosolic ROS and mROS production. In this sense, it has been stated that the interaction between Ca^2+^ and ROS is bidirectional, meaning that Ca^2+^ signaling is essential for ROS production, while ROS production is capable of regulating Ca^2+^ cell signaling [52]. It has been described in somatic cells that Ca^2+^ can regulate several ROS-generating enzymes, including cytochrome P450, cyclooxygenase, lipoxygenase, xanthine oxidase, and cell surface NADPH-oxidase [17], suggesting an influence of Ca^2+^ on intracellular ROS production under our experimental conditions. On the other hand, the increase in mROS production observed in this study may be a consequence of the drastic increase in mitochondrial Ca^2+^, which causes an uncoupling of the electron transport chain, by conformational changes in mitochondrial complexes I and III, leading to increased mROS production [53]. In our experimental conditions, it was observed that the increase in ROS production stimulated by the increase in intracellular Ca^2+^ overload by ionomycin was accompanied by the ΔΨm dissipation. This finding is consistent with a study in human spermatozoa, which reported that ionomycin-induced Ca^2+^ overload caused an increase in ROS production and a subsequent dissipation of ΔΨm, as a consequence of electron transport chain damage [21]. Specifically, increased ROS production causes the dissipation of ΔΨm due to its ability to induce the cytochrome c release from the mitochondria intermembrane space, leading to subsequent caspase cascade activation [54], which causes the dissipation of ΔΨm due to the alteration of complexes I and II of the electron transport chain [55]. Mitochondria are characterized by being the major organelle that generates ROS [56], which are produced as by-products of oxidative phosphorylation, especially in complexes I and III [57]. In addition, it maintains a dynamic relationship with Ca^2+^ [58] acting as a reservoir capable of regulating [Ca^2+^]_i_ through its uptake and release in response to fluctuations in cytosolic Ca^2+^. In this way, it modulates the increase in mitochondrial Ca^2+^ concentration ([Ca^2+^]_mt_), stimulating the tricarboxylic acid cycle and coupling energy demand with ATP production [59].

In this study, we also observed that the increase in ROS production caused by the stimulation of intracellular Ca^2+^ overload was not only accompanied by the dissipation of ΔΨm, but also caused a decrease in ATP levels. Previous reports indicated that the dissipation of ΔΨm, which is the driving force for ATP synthase, inhibited the production of ATP [60]. Direct ROS-induced damage to mitochondria and the cellular energy machinery also contributes to the decrease in energy production [61]. In addition, mitochondrial Ca^2+^ uptake has also been linked to the subsequent dissipation of ΔΨm [62]. Therefore, the increase in ROS production together with the stimulation of ionomycin-induced Ca^2+^ overload may contribute to the decrease in ATP production observed in our study. Considering the importance of the physiological levels of ROS, ΔΨm and ATP levels for the maintenance of sperm motility [63,64], the alteration of these parameters, i.e., the increase in ROS production [65,66,67], ΔΨm dissipation [68] and decreased ATP levels [68,69], may be closely related to the alteration of sperm motility observed under our experimental conditions. In this regard, it has been proposed that the uncoupling between the electron transport chain and oxidative phosphorylation, resulting from the excessive ROS production, entails dissipation of the ΔΨm necessary for the production of mitochondrial ATP and, therefore, for the maintenance of sperm motility [70]. Consistent with this evidence, it has been shown that the decrease in ATP production in mouse spermatozoa is closely related to the inhibition of the activity of complexes I, II, and III of the electron transport chain, caused by the increase in intracellular calcium concentration induced by ionomycin [71].

In addition, our results showed that the alteration in energy metabolism-related parameters resulted in decreased sperm viability and increased PS externalization. In relation to decreased sperm viability, the loss of plasma membrane integrity has been described as a hallmark of irreversible cell death [72], and the PS externalization is considered an early marker of cell death [73]. Our findings align with previous studies reporting that ionomycin can also induce the expression of other markers associated with apoptotic cell death, including caspase activation [74] and DNA fragmentation [21] in human sperm. This data, combined with the PS externalization observed in our study, suggests that ionomycin-induced intracellular Ca^2+^ overload may trigger cell death, contributing to sperm functional impairment. In addition, the changes in the plasma membrane observed under our experimental conditions may be a consequence of the cascade of lipid peroxidation caused by high levels of ROS [12], which exacerbate OS in the spermatozoa by covalently binding to nucleophilic protein centers present in the electron transport chain [13,75], increasing the flux of electrons that are subsequently associated with oxygen, generating O_2_^−^ [75]. Thus, a change in the permeability and fluidity of the plasma membrane drastically alters cellular integrity [76]. Interestingly, the bioenergetic function of mitochondria is not only key to the regulation of vital cell functions, but also plays an important role in cell death, as cells require energy to regulate the type of cell death [77]. Prolonged elevation of cytosolic Ca^2+^ alters Ca^2+^ homeostasis by activating the mitochondria-dependent apoptotic pathway [78]. In somatic cells, it has been described that under conditions of Ca^2+^ and ROS overload, the ΔΨm dissipation, the excessive production of ROS, the decoupling of oxidative phosphorylation, and the decrease in ATP production are the result of mitochondrial permeability transition pore (mPTP) opening, which subsequently leads to cell death [79]. Similarly, a study performed in human spermatozoa showed that the Ca^2+^ overload stimulated by ionomycin caused the mPTP opening, excessive ROS production, and ΔΨm dissipation, which is consistent with our results [21]. Therefore, we observed that the detrimental effects on the plasma membrane, energy metabolism, and sperm function following exposure to high concentrations of ionomycin may result from the activation of cell death pathways.

Our results can be explained by a dynamic interaction between the increase in ROS production and the intracellular Ca^2+^ overload stimulated by ionomycin. We propose that both the alterations in Ca^2+^ homeostasis and the increase in ROS levels influence each other, thereby amplifying the deleterious effects on sperm function and mitochondria-associated energy metabolism, leading to bioenergetics collapse, impaired sperm motility, plasma membrane alteration, and subsequent activation of sperm cell death.

It is important to note that this is an in vitro study, so true extrapolations to real-life reproductive scenarios cannot be made. Nevertheless, the observed effects of ionomycin-induced calcium overload may help to understand the damage to sperm function that occurs in contexts where excess ROS are produced, such as in the semen of infertile patients with high levels of seminal ROS, particularly those with MOSI, or in laboratory procedures that increase the production of these reactive species, such as sperm cryopreservation or in vitro gamete manipulation, which includes centrifugation and washing processes.

## 5. Conclusions

Ca^2+^ overload impairs the bioenergetic function of mitochondria in human spermatozoa, which is associated with a decrease in sperm quality and induction of cell death. This may help to understand the mechanisms underlying oxidative stress-related damage to sperm function in scenarios with elevated ROS levels, such as infertile patients with MOSI, or laboratory procedures such as cryopreservation and/or gamete manipulation that increase production of these reactive species.

## Figures and Tables

**Table 1:** Effect of intracellular Ca^2+^ overload on sperm motility in human spermatozoa.

Sperm Motility Patterns		Ionomycin (µmol/L)		
	Untreated Control	0.1	1	10
Progressive Motility (%)	30.6 ± 10.5	39.0 ± 9.7	18.9 ± 10.2	0 ± 0.0 *
Non-Progressive Motility (%)	47.2 ± 8.5	42.4 ± 9.9	15.5 ± 15.8 *	0 ± 0.0 **
Immotile (%)	22.2 ± 18.4	18.6 ± 11.3	65.5 ± 26 **	100 ± 0.0 ***

Selected human spermatozoa were exposed to 0.1, 1, and 10 µmol/L of ionomycin for 1 h at 37 °C, in order to induce intracellular Ca^2+^ overload. An untreated control was included. The results correspond to the mean ± SD of five different experiments. * *p* < 0.05, ** *p* < 0.01, *** *p* < 0.001, N = 5.

## Data Availability

The data supporting the conclusions of this article are available from the corresponding author upon reasonable request.
